# A Double Dissociation between Conscious and Non-conscious Priming of Responses and Affect: Evidence for a Contribution of Misattributions to the Priming of Affect

**DOI:** 10.3389/fpsyg.2017.00453

**Published:** 2017-03-27

**Authors:** Florian Goller, Shah Khalid, Ulrich Ansorge

**Affiliations:** ^1^Faculty of Psychology, University of ViennaVienna, Austria; ^2^Institute of Cognitive Science, University of OsnabrückOsnabrück, Germany

**Keywords:** flanker task, masking, non-conscious processing, misattributions of affect, conflict

## Abstract

Studies have demonstrated conscious and non-conscious priming of responses and of affect. Concerning response priming, presenting a target-related (congruent) distractor prior to a target typically facilitates target responses. This facilitation – the response-priming effect – is observed in comparison to a less related (incongruent) distractor. An incongruent distractor would interfere with the required response to the target. This response-priming effect is found with both conscious distractors, of which participants are aware, and non-conscious distractors, of which participants are not aware. In partly related research, distractors have also yielded affective priming effects on the evaluations of task-unrelated neutral symbols that followed the target: In comparison to the congruent condition, participants evaluated a neutral symbol presented after an incongruent distractor-target sequence as more negative. This affective priming effect was sometimes ascribed to the participants’ misattributions of distractor-target conflict to the unrelated neutral symbols. Here, we set out to test this possibility. If the misattribution explanation of affective priming holds true, affective priming would be stronger with non-conscious than with conscious distractors: Mostly the non-conscious distractors would mask distractor-target conflict as the true affect-origin and, therefore, invite participants’ misattribution of the primed affect to the neutral symbol in temporal vicinity. In contrast, only with conscious distractors, participants would be aware of distractor-target conflict as the true affect-origin and should, therefore, be better able to attribute their affective responses to the distractor-target relationship itself. In three experiments, we confirmed this prediction of a stronger affective priming effect in non-conscious than conscious distractor conditions, while at the same time showing conscious response-priming effects to even exceed non-conscious response-priming effects. Together, these results amount to a double dissociation between affective priming, being stronger with unconscious distractors, and response priming, being stronger with conscious distractors. This double dissociation supports the misattribution explanation and makes clear that the amount of distractor-elicited response conflict alone does not account for the amount of affective priming. Moreover, the participants’ unawareness of the distractors is critical for the amount of affective priming of neutral symbols in temporal vicinity.

## Introduction

Have you ever snapped at a friend for no apparent reason? Have you ever felt sad without knowing why? These examples illustrate that humans can experience negative (or positive) affective responses without an awareness of the objects that elicited the affective responses. In the present article, affective responses are defined as the actual positive or negative impressions that are elicited by an object, and the true affect-origin is defined as the object that triggered these impressions. As our examples imply, humans are sometimes uncertain about the true origins of their affective responses, an observation in line with emotion theories. For example, Arnold (1960) explained that emotions can be triggered without an awareness of their true origins. In general, Arnold believed that emotions are based on appraisals of objects or events as positive or negative. These appraisals correspond to what we called positive and negative affective responses. In the case of an automatic emotion generation, Arnold argued that the appraisal process is hidden from introspection, meaning that participants do not have to become aware of the true affect-origins. In more recent emotion theories, this claim was repeated. A number of researchers claimed that feelings that are initially elicited are only subsequently attributed to an object as its origin (Schachter and Singer, 1962; Weiner, 1986). For instance, in the feeling-as-information theory (Schwarz, 1990; see also Ortony et al., 1990), the feeling is the first state of awareness that informs a person about her own appraisal of the object that triggered the feeling–that is, before her feeling, this person would not know whether or not s/he liked an object (i.e., evaluated an object as positive or negative).

Considered from the perspective of such emotion theories, a person’s uncertainty about the true affect-origin in general, and the unawareness of the true affect-origin in particular, could be favorable side conditions of a misattribution of affective responses to alternative objects than the true affect-origin (cf. Zajonc, 1968; Murphy and Zajonc, 1993; Payne et al., 2010). Over the course of three experiments, we tested this hypothesis. We primed affective responses by distractors of which our participants were unaware (i.e., with visually masked distractors) or aware (i.e., with clearly visible distractors) and tested whether participants’ unawareness of the masked distractors as the true affect-origins is a favorable precondition for misattributions of affective responses away from the masked distractors and toward neutral objects in close temporal vicinity.

In our experiments, we used response priming to elicit affective responses (cf. Fritz and Dreisbach, 2013; see also van Steenbergen et al., 2010). In each trial of our experiments, participants had to discriminate between the orientations of a centrally presented target. If the current trial’s target was a square, participants had to press one key, and if the target was a diamond, they had to press another key. Prior to the central target, we presented two distractors at target-adjacent positions. In congruent trials, the distractor was of the same shape as the target and, thus, indicated the same response as the target. In incongruent trials, the distractor was of the alternative shape as compared to the target and, thus, led to response conflict between distractor and target. In this experimental situation, past research has demonstrated that, in comparison to congruent distractors, incongruent distractors delay the response to the target. This response-priming effect has been found with conscious distractors, of which participants were aware (Eriksen and Eriksen, 1974; Gratton et al., 1988), and with non-conscious distractors, of which participants were not aware (Schwarz and Mecklinger, 1995). However, with non-conscious distractors, this response-priming effect is typically weaker than with conscious distractors (Tapia et al., 2010).

Critically, in incongruent conditions a response conflict also elicits more negative affective responses than in congruent conditions where a response conflict is absent. This affective priming effect is measured in evaluations of neutral symbols that follow the distractor and the target in close temporal vicinity. Affective priming of neutral symbols through response conflict has hitherto been demonstrated with conscious distractors in a Stroop paradigm only (Fritz and Dreisbach, 2013). In their experiment, participants showed more negative ratings of a neutral Chinese symbol if presented after an incongruent Stroop (a mismatch between a color word and the print color of the word, e.g., GREEN printed in red) than after a congruent (GREEN printed in green) Stroop. Studies showing that affective priming of neutral objects have sometimes been ascribed to misattributions of affective responses away from the eliciting stimulus and toward the neutral symbol or object (Payne et al., 2005).

Here, we tested this misattribution explanation of affective priming. If misattributions account for affective priming, affective priming could be stronger with non-conscious than with conscious affect-origins. The reason is that with conscious affect-origins, participants are well aware of degree of the distractor-target incongruence as the true affect-origin. This makes it less likely that the participants misattribute the same affective response elicited by the distractor-target pair also to a different neutral symbol following the distractor and target (cf. Oikawa et al., 2011). In contrast, with non-conscious affect-origins, participants are not aware of the distractor-target incongruence as the true affect-origin. This could foster the misattribution of the distractor-elicited affective response to the neutral symbol that follows the distractor and target. In other words, affective priming effects for a neutral symbol could be stronger with non-conscious distractors than with conscious distractors, despite the fact that response priming could be stronger with conscious distractors than with non-conscious distractors. Together, these expectations thus amount to the prediction of a double dissociation between response priming and affective priming by non-conscious and conscious distractors.

## Experiment 1

The aim of our study was to assess the influence of conscious and non-conscious conflict on the evaluation of a neutral symbol. Conflict was elicited by target-distractor congruence in a flanker task (Eriksen and Eriksen, 1974). We chose a flanker task because non-conscious conflict is established very well in this task (Schwarz and Mecklinger, 1995; Tapia et al., 2010) and is less controversial than with other congruence tasks (e.g., Stroop tasks, see Kouider and Dupoux, 2004).

To measure affective priming, participants additionally rated a neutral Chinese symbol as positive or negative at the end of some trials. If target-distractor incongruence elicits more negative affective responses than target-distractor congruence and if these affective responses are (sometimes) misattributed, we expected more negative evaluations of the symbols in incongruent than congruent trials. Furthermore, a failure to perceive the true affect-origin could increase the likelihood of misattributions, resulting in more frequent affect misattributions following non-conscious than conscious distractors (cf. Murphy and Zajonc, 1993).

At the end of each block, we also measured the participants’ awareness of the distractors. This was done to ensure that our participants were indeed not aware of the masked, non-conscious distractors, as well as to ensure that our participants were aware of the clearly visible, conscious distractors. In this awareness test, we applied the chance-level performance criterion of unawareness (Klotz and Neumann, 1999; Schmidt and Vorberg, 2006). In each trial of the awareness test, participants had to decide if they though the current prime was a square or a diamond. In this awareness test, chance performance means that the participants were unable to successfully discriminate between the distractor shapes and, thus, that the participants were unaware of the distractors. In contrast, above chance performance could mean that (1) masking was not perfect, such that on some trials the distractors were visible and participants were aware of them, or that (2) there distractors are processed in a consciousness-independent way, allowing above chance performance without conscious access to them. Despite these limitations, we expected chance-level performance in the non-conscious condition but better than chance performance in the conscious condition.

### Methods

#### Participants

Twenty-eight participants (15 female, 13 male, *M*_Age_ = 24.05, *SD*_Age_ = 8.64) were tested. We calculated this sample size and all following sample sizes by using G^∗^Power (Faul et al., 2009) under the assumption of a small to medium effect size and a statistical power of 90%. Two participants were excluded due to more than 20% errors. Here and in all following experiments, participants received course credit, were right-handed, had normal or corrected-to-normal vision, and reported no prior experience with Chinese symbols. Prior to all experiments, all participants gave written consent and were informed that participation and data collection were fully anonymous. Participants could withdraw at any time during the experiment without any further consequences. All studies were conducted in accordance with the Declaration of Helsinki (revised, 1983) and the guidelines of the Faculty of Psychology, University of Vienna. We further followed the Austrian Universities Act, 2002 (UG2002) – which was active at the time of the experiments – which required only medical universities to appoint ethics committees for clinical testing, application of medical methods and applied medical research. Therefore, no additional ethical approval was sought.

#### Apparatus and Stimuli

The experiment was programmed and controlled using Matlab 7.7.0 (The MathWorks inc., Natick, MA, USA) and the Psychophysics Toolbox (Brainard, 1997; Pelli, 1997). Viewing distance was stable at 57 cm, supported by a chin and forehead rest. Responses were manual key presses with the left versus right index finger on a keyboard. Target responses were given through the keys ‘f’ and ‘j’. Targets and distractors (1.5° × 1.5°) were squares and diamonds (Klotz and Neumann, 1999). To decrease participants’ awareness of the distractors, in the non-conscious condition, we used circular metacontrast masks that were neutral with respect to the response-relevant angular target shapes (2° × 2°). A fixation-cross (0.5° × 0.5°) was displayed at screen center. Targets appeared always on the screen’s vertical meridian, 6.2° equally likely above or below the fixation-cross. Distractors and masks were placed equally distant (2.2°) left and right of the target. In half of the trials, target and distractors were of the same shape (congruent trials). In the other half of the trials, target and distractors were of different shapes (incongruent trials). All stimuli were colored black (CIE Lab: 0.9/-0.3; 0.8 cd/m^2^) and were presented against a gray background (CIE Lab: 0.9/-8.4; 31.1 cd/m^2^).

Randomly, one third of the trials were followed by a rating task with a black Chinese symbol (4° × 4°) at screen center. The symbols were randomly selected from an online English-Chinese dictionary^1^. In a pre-study, fresh participants rated a total set of 700 symbols on valence, arousal, and complexity. To rule out *a priori* evaluation differences between the symbols used, we selected symbols with moderate ratings on these dimensions. Also, by randomization of the symbols across trials, each specific symbol was equally likely used in congruent and incongruent trials. We verified this assumption by conducting chi-square tests of the occurrence of each individual symbol on each level of the variables consciousness (conscious vs. non-conscious) and congruence (congruent vs. incongruent). None of these tests were significant, all χ^2^< 2.59, all *p* < 0.108.

#### Procedure

**FIGURE 1:**
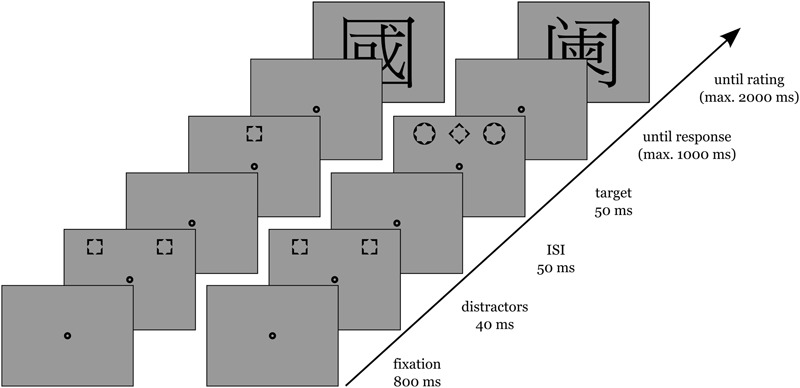
**Example trials of Experiments 1, 2, and 3, with time flowing from bottom to top.** The left side depicts a congruent conscious trial, the right side depicts an incongruent non-conscious trial. Note that only in one third of the trials, participants had to rate a Chinese symbol. The figure is not drawn to scale.

After the fixation display (800 ms), distractors were added (40 ms), and then, with an inter-stimulus interval (ISI) of 50 ms, a target was shown for 100 ms (see also **Figure 1**). Participants were first tested in the non-conscious block, and afterward in the conscious block. The order of blocks was kept constant to ensure participants’ minimal awareness of the non-conscious distractors prior to the distractor-awareness test at the end of the experiment (see below). Participants pressed the left or right key, with each key mapped to a specific target shape (counterbalanced across participants). An on-screen feedback informed about too slow (>1 s) or erroneous responses. In one third of the trials, following target discrimination, participants rated the valence of a Chinese symbol as either negative (left key) or positive (right key). This mapping was the same for all participants since the dominant hand seems to be associated with positive concepts (Casasanto, 2009). In total, the experiment consisted of 768 trials.

In a separate part at the end of the experiment, participants’ awareness of the distractors was assessed. Participants were informed about the presence and identities of the distractors, and their task was to discriminate and report distractor identities. Across trials, stimulus-to-response (S-R) mapping varied randomly (announced via presentation of a post-target onscreen mapping rule). S-R mapping rules varied from trial to trial and were only specified after the targets to ensure that the awareness test was exclusive – that is, that the awareness test was only sensitive for the conscious perception of the primes (cf. Reingold and Merikle, 1988). If we would have used a fixed S-R mapping rule for different distractor shapes, which the participants knew in advance of the distractors, awareness-independent response specification by the masked distractors could have contributed to the number of correct responses in the awareness test (see Experiment 4 of Neumann and Klotz, 1994). This would have yielded a non-exclusive measure of distractor awareness – that is, an “awareness score” that does not deserve this label as it would have reflected both awareness-dependent and awareness-independent contributions of distractor processing. As only awareness was to be measured by this test, awareness-independent contributions had to be ruled out. In total, the distractor visibility assessment block consisted of 320 trials. In general, independence of awareness was to be ensured by participants’ chance-level performance in this awareness-test block. However, one problem with this kind of criterion is that a non-significant *p*-value of a *t*-test against chance performance (50%) or zero does not inform us about whether truly no effect was found or our test was insensitive to the effect (see Dienes, 2014). To resolve this problem, we additionally conducted Bayesian Factor (BF) analysis of the corresponding *t*-tests (Rouder et al., 2009), with an R scale of 1. Here we reported the scaled JZS Bayes factor values. They indicate the relation between the probabilities of the data being in favor of the null relative to being in favor of the alternative hypothesis (or vice versa). On the basis of Jeffreys (1961) convention, a Bayes factor greater than 3.00 was considered as a substantial evidence in favor of the null or the alternative hypothesis accordingly (Dienes, 2011).

### Results

#### Reaction Times (RTs)

**FIGURE 2:**
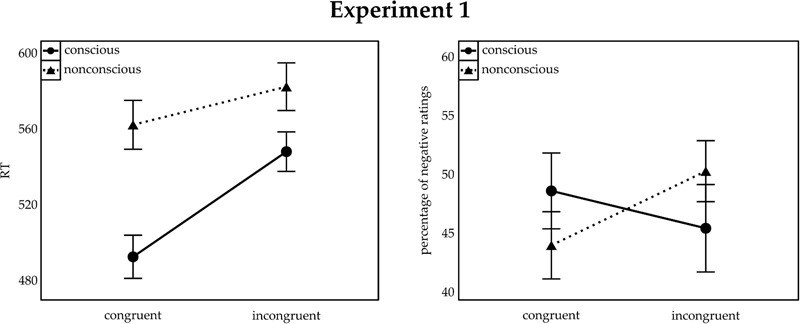
**Mean reaction time (RT) to target shapes (Left)** and percentage of negative ratings of neutral symbols **(Right)** for Experiment 1. Congruent and incongruent trials are indicated on the *x*-axis. The separate lines illustrate conscious (solid lines) and non-conscious (dashed lines) trials. Error bars represent standard error of the mean (SEM).

Trials with erroneous responses (8.80%) were analyzed separately (see below). Mean correct reaction times (RTs) were subjected to a repeated-measurements analysis of variance (ANOVA), with the within-participant variables consciousness (conscious; non-conscious) and congruence (congruent; incongruent). Where appropriate, degrees of freedom were Greenhouse-Geisser corrected. For better transparency, the uncorrected degrees of freedom but the corrected *p*-values are reported. **Figure 2** (left panel) illustrates the results.

Besides main effects of consciousness, *F*(1,25) = 73.55, *p* < 0.001, $\eta_{p}^{2}$ = 0.75, and congruence, *F*(1,25) = 102.12, *p* < 0.001, $\eta_{p}^{2}$ = 0.80, we also found an interaction, *F*(1,25) = 40.86, *p* < 0.001, $\eta_{p}^{2}$ = 0.62. In the conscious block, mean RT was lower in congruent (492 ms) than incongruent trials (548 ms), *t*(25) = -12.73, *p* < 0.001, *d* = 3.53. The same was true of the non-conscious block (congruent: 562 ms; incongruent: 582 ms), *t*(25) = -4.08, *p* < 0.001, *d* = 1.13, but the congruence effect (incongruent RT minus congruent RT) was significantly larger in the conscious (56 ms) than in the non-conscious (20 ms) block, *t*(25) = 6.39, *p* < 0.001, *d* = 1.77.

Since the to-be-rated symbol was only shown in a third of all trials, we conducted a separate, complementary analysis of the RTs on only those trials in which a Chinese symbol had to be rated. The results were essentially the same as above. We had two significant main effects and an interaction (all *F* > 20.13, all *p* < 0.001). Again, RTs were faster in congruent than incongruent trials in both the conscious (489 ms vs. 550 ms), *t*(25) = -10.07, *p* < 0.001, *d* = 2.79), and the non-conscious block (563 ms vs. 582 ms), *t*(25) = -2.55, *p* < 0.001, *d* = 0.71). Additionally, the congruence effect was larger in the conscious (61 ms) than the non-conscious (19 ms) block, *t*(25) = 4.49, *p* < 0.001, *d* = 1.24.

#### Error Rates (ERs)

The arcsine transformed error rates (ERs) were subjected to a similar ANOVA as was used for RTs. A main effect of congruence, *F*(1,25) = 40.30, *p* < 0.001, $\eta_{p}^{2}$ = 0.62, interacted with consciousness, *F*(1,25) = 39.74, *p* < 0.001, $\eta_{p}^{2}$ = 0.61. No main effect of consciousness was found, *F* = 0.27. In the conscious block, we found higher ERs in incongruent (11.54%) than congruent (4.70%) trials, *t*(25) = -7.39, *p* < 0.001, *d* = 2.05. No such difference was found in the non-conscious block (7.05% vs. 7.51%), *t*(25) = -1.23, *p* = 0.230, *d* = 0.34.

#### Ratings of the Chinese Symbols

From all correct flanker-task trials, we computed the probabilities of negative ratings of the Chinese symbols for each participant and combination of consciousness and congruence. Across all conditions, the probability of a negative rating was not significantly different from 50%, *t*-test against 50%: *t*(25) = 1.27, *p* = 0.215, *d* = 0.35, *BF* = 3.10 in favor of the null hypothesis. The same was true for separate analyses of the non-conscious, *t*(25) = 1.16, *p* = 0.124, *d* = 0.45, *BF* = 3.50 in favor of the null hypothesis, and the conscious, *t*(25) = 1.02, *p* = 0.316, *d* = 0.28, *BF* = 4.03 in favor of the null hypothesis, blocks. This indicates that on average symbols were rated relatively neutral, meaning that no general *a priori* trend for negative or positive ratings of the Chinese symbols was present.

A repeated-measurements ANOVA of the arcsine transformed probabilities of negative ratings revealed an interaction between congruence and consciousness, *F*(1,25) = 9.75 *p =* 0.004, $\eta_{p}^{2}$ = 0.28. No significant main effects were found, both *F* < 0.61, both *p* < 0.249. **Figure 2** (right panel) illustrates the results. In the non-conscious block, participants rated the symbols more often as negative in incongruent trials (50.00%) than congruent trials (43.65%), *t*(25) = 3.46, *p* = 0.002, *d* = 0.96, *BF* = 17.33 in favor of the alternative hypothesis. Most notably, no such difference was found in the conscious block (congruent: 48.27%; incongruent: 45.09%), *t*(25) = 1.27, *p* = 0.216, *d* = 0.35, *BF* = 3.10 in favor of the null hypothesis.

#### Distractor Awareness

Awareness of the distractors was assessed by *d’* (Green and Swets, 1966/1974). Scores of *d*’ were obtained from direct calculation of hit rates and false alarm rates. For the calculation of *d’*, diamonds counted as signals and squares as noise. Here, *d*’ is the *z*-transformed false alarm rate subtracted from the *z*-transformed hit rate. This index becomes zero in the case of chance performance (i.e., unawareness), and it can infinitely increase with ever increasing discrimination performance. Performance was above chance in conscious trials (*d’* = 1.05), *t*-test against zero: *t*(25) = 5.32, *p* < 0.001, *d* = 1.47, *BF* = 1435.32 in favor of the alternative hypothesis, and not different from chance in non-conscious trials (*d’* = 0.08), *t*-test against zero: *t*(25) = 0.78, *p* > 0.249, *d* = 0.22, *BF* = 4.94 in favor of the null hypothesis.

To exclude the possibility of a response bias, or more importantly, different response biases in the conscious and non-conscious trials, we also analyzed the criterion β, which is calculated as the ratio between the likelihood of choosing one response in signal trials and the likelihood of choosing the same response in a noise trial (Stanislaw and Todorov, 1999). This ratio becomes 1 if participants favor neither response over the other. The β values were not significantly different in conscious (β = 1.07) and non-conscious (β = 1.18) trials, *t*(25) = 0.41, *p* > 0.249, *d* = 0.11, *BF* = 6.10 in favor of the null hypothesis.

### Discussion

We see from the chance performance in the distractor-awareness tests of the non-conscious block that participants were not aware of the masked distractors. Although non-conscious distractors elicited a response conflict, as shown in the RTs, the true origin of this conflict in the target-distractor relation, therefore, remained unknown to the participants. This should have led to an active search for an alternative origin of the conflict-elicited affect–that is, an alternative origin to the distractor-target congruence versus incongruence. In line with this expectation, neutral Chinese symbols following the targets were judged more often as negative following a non-conscious incongruent than following a non-conscious congruent distractor. Participants evidently misattributed the origin of their negative affect to a neutral stimulus in close temporal vicinity: the Chinese symbol.

Crucially, in line with a supportive role of unawareness of the true affect-origins for affect misattribution, no affect misattributions were found following conscious distractors: With conscious congruent and incongruent distractors, equal proportions of negative and positive judgments were observed, although the RTs were clearly influenced by conscious target-distractor congruence/incongruence, too: Conscious distractors produced an even stronger effect on RTs than non-conscious distractors (for a similar finding see Tapia et al., 2010). Together with the results of the non-conscious block, the data amount to a double dissociation between response priming and affective priming. While response priming was stronger in conscious than non-conscious blocks, affective priming was stronger in non-conscious than conscious blocks. This means that the difference in terms of affective priming was not simply due to a stronger response-congruence effect in the non-conscious block.

A stronger misattribution effect with non-conscious than conscious distractors is in line with our predictions (see also Murphy and Zajonc, 1993, for an exposure-elicited affect misattribution). However, an entire lack of affect misattributions following conscious distractors is at variance with affect misattributions following conscious Stroop trials in the study of Fritz and Dreisbach (2013). One decisive difference between the present study and the one by Fritz and Dreisbach (2013) was that participants did not respond to the Stroop target itself but only had to judge the Chinese symbol following the Stroop target. In contrast, in the rating trials of the present experiment, participant first had to respond to the target of the flanker task and then rated the Chinese symbol following the target. Therefore, the use of no-go trials prior to the symbol ratings in the study of Fritz and Dreisbach (2013) was maybe critical for affect misattributions in conscious target-distractor trials. For instance, responding correctly to the flanker targets in the incongruent trials of the present study could have changed our participants’ evaluation of conflict from negative to more positive (Schouppe et al., 2015). Therefore, our participants could have felt more positive about their successful response to a target following a conscious incongruent distractor than the participants of Fritz and Dreisbach (2013). Additionally, our participants might have felt less positive about their correct responses to non-conscious incongruent distractors simply because our participants would have failed to register the non-conscious incongruent distractor as a challenge in the first place. Consequently, only the present conscious incongruent distractors, but not the non-conscious incongruent distractors might have prompted positive feelings of success that counteracted the negative affective responses (and their misattributions to the symbols).

One last point needs also mentioning: Our participants might have associated the response key with the valence conveyed by the distractor-target pair (see Gawronski and Ye, 2014), meaning that a repetition of the response key (e.g., right key for square and the same right key for a positive evaluation) might have influenced the ratings in some cases. To clarify these issues, we conducted Experiment 2, in which our participants rated the Chinese symbols in go and no-go trials, the former replicating our Experiment 1, the latter being more similar to the experimental setup of Fritz and Dreisbach (2013).

## Experiment 2

We used a go/no-go flanker task, where participants had to react to a target shape (e.g., a square, the go target) and had to withhold their response if the alternative target shape was presented (e.g., a diamond, the no-go target). Shortly before the target, the distractors were presented in the same manner as in Experiment 1. A no-go distractor is incongruent to the go target and accordingly delays the go response (for congruence effects with non-conscious distractors in the go/no-go task see Experiment 5 of Ansorge, 2004). We expected an RT congruence effect in the go trials, and affect misattributions based on congruence versus incongruence in go and no-go trials. If the participants’ unawareness of the distractors facilitated affect misattributions, we expected more misattributions following non-conscious than conscious distractors. However, if in Experiment 1 overt correct responses to conscious incongruent distractors changed the evaluations and prevented a misattribution effect, we expected more misattributions following conscious distractors in the present no-go than go trials.

### Methods

#### Participants

Twenty-eight participants were tested. Two participants were *post hoc* excluded due to more than 20% errors in the flanker task. The final sample consisted of 17 females and 9 males (*M*_Age_ = 23.46, *SD*_Age_ = 3.59).

#### Apparatus and Stimuli

These were the same as in Experiment 1, with two exceptions. To reduce visual crowding and task difficulty, we reduced distractor and target eccentricities to 4.5° (6.2° in Experiment 1), and slightly increased target-distractor distances to 2.5° (2.0° in Experiment 1).

#### Procedure

The sequence of events in a trial was as in Experiment 1 (see **Figure 1**), but the task was changed. If the target was a diamond (or a square, counterbalanced across participants), participants pressed the spacebar. Otherwise, they had to withhold their response and to wait until the next trial started. There was a fixed time window of 1 s for participants to give their answer (in go trials) or to wait (in no-go trials). Otherwise the task and the procedure were the same as in Experiment 1.

### Results

#### Reaction Times (RTs)

**FIGURE 3:**
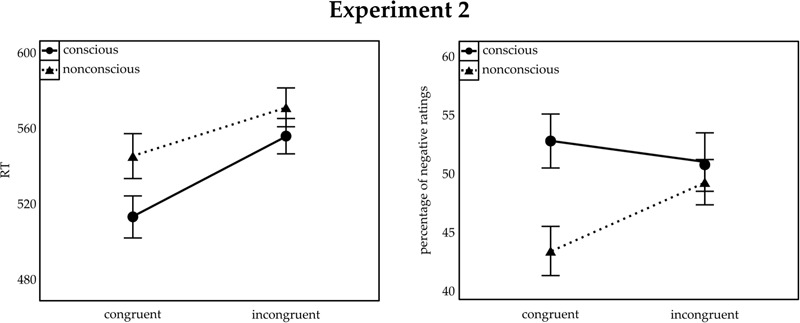
**Mean RT to target shapes (Left)** and percentage of negative ratings of neutral symbols **(Right)** for Experiment 2. Congruent and incongruent trials are indicated on the *x*-axis. The separate lines illustrate conscious (solid lines) and non-conscious (dashed lines) trials. Error bars represent SEM.

All error trials (7.55%) were excluded and analyzed separately (see below). Main correct go-trial RTs were subjected to a repeated-measurements ANOVA, with the within-participant variables consciousness (conscious; non-conscious) and congruence (congruent; incongruent), analogous to Experiment 1. The results are shown in **Figure 3** (left panel). Besides significant main effects of consciousness, *F*(1,25) = 11.14, *p* = 0.003, $\eta_{p}^{2}$ = 0.31, and congruence, *F*(1,25) = 54.58, *p* < 0.001, $\eta_{p}^{2}$ = 0.69, we also found an interaction between these variables, *F*(1,25) = 8.19, *p* = 0.008, $\eta_{p}^{2}$ = 0.25. Similar to Experiment 1, RTs were faster in congruent than incongruent trials for both the non-conscious (congruent: 547 ms; incongruent: 572 ms), *t*(25) = 5.08, *p* < 0.001, *d* = 1.41, and the conscious (congruent: 517 ms; incongruent: 558 ms), *t*(25) = 7.25, *p* < 0.001, *d* = 2.01) block. Again, the RT congruence effect (incongruent RT minus congruent RT) was significantly larger in conscious (41 ms) than non-conscious (25 ms) blocks, *t*(25) = 2.86, *p* = 0.008, *d* = 0.79. An analysis restricted to only the trials where a symbol was rated, yielded essentially the same results.

#### Error Rates (ERs)

The arcsine transformed mean ERs were subjected to a similar ANOVA, with the additional variable of task (go; no-go). The main effects of task, *F*(1,25) = 11.84, *p* = 0.002, $\eta_{p}^{2}$ = 0.32, and congruence, *F*(1,25) = 41.36, *p* < 0.001, $\eta_{p}^{2}$ = 0.62, as well as the interactions between consciousness and congruence, *F*(1,25) = 4.83, *p* = 0.037, $\eta_{p}^{2}$ = 0.16, and task and congruence, *F*(1,25) = 11.26, *p* = 0.003, $\eta_{p}^{2}$ = 0.31, are best explained by looking at the interaction between all three factors, *F*(1,25) = 8.62, *p* = 0.007, $\eta_{p}^{2}$ = 0.26. No other main effects or interactions were found, all non-significant *F* < 0.07.

To explore the significant three-way interaction, we conducted separate ANOVAs for the go and no-go trials. In the go trials, we obtained a significant main effect of congruence, *F*(1,25) = 38.36, *p* < 0.001, $\eta_{p}^{2}$ = 0.61, and an interaction with consciousness, *F*(1,25) = 9.53, *p* = 0.005, $\eta_{p}^{2}$ = 0.28. No main effect for consciousness was found, *F* = 0.10. In the conscious block, ERs were higher in incongruent (11.18%) than congruent (2.69%) trials, *t*(25) = 5.73, *p* < 0.001, *d* = 1.59. In the non-conscious block, a similar pattern was found (incongruent: 8.71%; congruent: 4.66%), *t*(25) = 3.58, *p* = 0.001, *d* = 0.99. Analogous to the RTs, the ER congruence effect was larger in the conscious (8.48%) than in the non-conscious (4.04%) block, *t*(25) = 3.09, *p* = 0.005, *d* = 0.86.

In the no-go trials, only a main effect of congruence was found, *F*(1,25) = 11.37, *p* = 0.002, $\eta_{p}^{2}$ = 0.31, indicating a higher ER in incongruent (4.43%) than congruent (2.84%) trials. No other effects were found for the no-go trials, all non-significant *F* < 0.02.

#### Ratings of the Chinese Symbols

The analysis was based on correct flanker-task trials only. No overall bias toward negative (or positive) evaluations was found, *t*-test against 50%: *t*(25) = -0.49, *p* > 0.249, *d* = 0.13, *BF* = 5.89 in favor of the null hypothesis. The same holds almost true if tests were conducted separately for the non-conscious block, *t*(25) = -1.76, *p* = 0.090, *d* = 0.49, *BF* = 1.60 in favor of the null hypothesis–this value is less than 3, and therefore not substantial evidence–, and the conscious block, *t*(25) = -0.52, *p* > 0.249, *d* = 0.14, *BF* = 5.81 in favor of the null hypothesis. A repeated-measurements ANOVA, with the same variables as in the ERs, revealed a main effect of consciousness, *F*(1,25) = 4.84, *p* = 0.037, $\eta_{p}^{2}$ = 0.16, and an interaction between consciousness and congruence, *F*(1,25) = 9.16, *p* = 0.006, $\eta_{p}^{2}$ = 0.27. In the non-conscious block, participants rated the symbols less often as negative in congruent (43.13%) than incongruent (49.00%) trials, *t*(25) = 2.88, *p* = 0.008, *d* = 0.80, *BF* = 4.88 in favor of the alternative hypothesis. There was no such difference in the conscious block (congruent: 52.51%; incongruent: 50.71%), *t*(25) = 1.46, *p* = 0.157, *d* = 0.41, *BF* = 2.45 in favor of the null hypothesis. Furthermore, we found a strong trend toward a main effect of task, *F*(1,25) = 4.06, *p* = 0.055, $\eta_{p}^{2}$ = 0.14, with a lower proportion of negative ratings in go trials (47.16%) than no-go trials (50.51%). A follow-up Bayesian *t*-test with a Bayes factor of 1.84 indicates, however, that this trend is not entirely conclusive. No other effects were found, all non-significant *F* < 3.13, all *p* > 0.089. **Figure 3** (right panel) illustrates the results.

#### Distractor Awareness

Distractor discrimination was above chance in the conscious trials (*d’* = 1.32), *t*-test against zero: *t*(25) = 6.96, *p* < 0.001, *d* = 1.93, *BF* = 68177.77 in favor of the alternative hypothesis, and not different from chance in the non-conscious trials (*d’* = 0.07), *t*(25) = 0.64, *p >* 0.249, *d* = 0.18, *BF* = 5.43 in favor of the null hypothesis. No difference in the response bias was found between the conscious trials (β = 1.06) and non-conscious trials (β = 0.99), *t*(25) = 1.20, *p =* 0.240, *d* = 0.33, *BF* = 3.35 in favor of the null hypothesis.

### Discussion

Experiment 2 confirmed that affect misattributions were restricted to the non-conscious distractors. With conscious distractors, congruence-elicited affect misattributions were missing in go and no-go trials. The results are in line with a critical role of unawareness for affect misattributions to the neutral symbols (as in Experiment 1). In contrast, successful conscious discrimination of the incongruent distractors just prior to a symbol was not responsible for lacking affect misattributions following conscious distractors. Otherwise, we would have found a difference between the ratings of the symbols in go and no-go trials.

We also observed some evidence for another congruence-independent type of affect misattribution to neutral symbols that was based on events of which the participants were aware: a trend in the ANOVA toward more negative ratings of the symbols in no-go than go trials. To note, the Bayesian test revealed that this trend is not entirely convincing. However, if this effect were real, these affective responses probably reflected “distractor devaluation,” which denotes that (no-go) stimuli requiring to be ignored or associated with (response) inhibition are evaluated more negatively than (go) targets (Raymond et al., 2003; Fenske et al., 2005). Because this effect, if it existed, was due to the no-go targets but measured in the evaluations of the symbols, devaluation would have been due to affect misattribution, too. Yet, participants were aware of the no-go status of the targets as indicated by their low error rates in no-go trials, and, thus, participants must have been aware of the true affect-origins in these cases. Therefore, even in the present study not all affect misattributions would have depended on the participants’ unawareness of the true affect-origins (see also Payne et al., 2005; Fritz and Dreisbach, 2013).

## Experiment 3

Experiment 2 essentially replicated the results of Experiment 1. However, both experiments shared one major caveat: Participants were first tested in the non-conscious block and afterward in the conscious block. We used a fixed block order to minimize participants’ distractor awareness or distractor suspicion during the non-conscious block. Therefore, we cannot be sure whether our effects were truly caused by the manipulation of consciousness or whether block order was (partly) responsible for our results. Maybe participants were just more used to the method in the later occurring conscious blocks than in the earlier occurring non-conscious blocks. For instance, if the participants had learned to actively ignore the unwanted affective priming influence of the target-distractor congruence on the symbol ratings, this influence would have had a greater effect on the affective priming effect in the conscious than in the non-conscious block.

To address this issue, we conducted a control experiment in which we replicated Experiment 2, but manipulated distractor consciousness between participants, not within participants. We decided to use a between-participants design to rule out all possible transfer effects between the conscious and non-conscious blocks. To state the results of Experiment 3 right from the outset: We essentially mirrored the results of Experiment 2. Hence, our results truly stem from the manipulation of distractor awareness.

### Methods

#### Participants

Thirty-six participants were tested, 18 in the conscious condition (12 females and 6 males, *M*_Age_ = 21.00, *SD*_Age_ = 1.88) and 18 in the non-conscious condition (16 females, 2 males, *M*_Age_ = 22.00, *SD*_Age_ = 6.65).

#### Apparatus, Stimuli, and Procedure

These were the same as in Experiment 2, with the notable difference that participants were tested only in the conscious or only in the non-conscious block.

### Results

#### Reaction Times (RTs)

**FIGURE 4:**
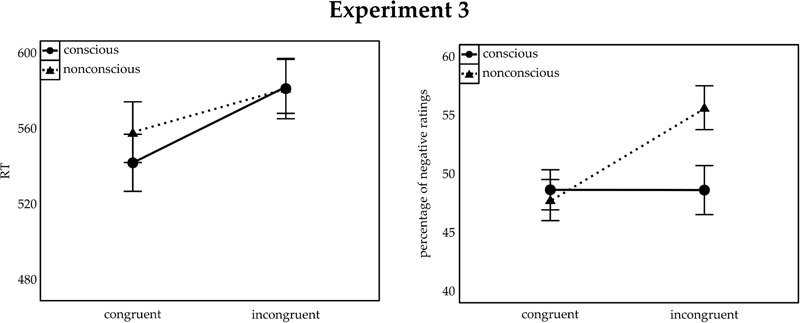
**Mean RT to target shapes (Left)** and percentage of negative ratings of neutral symbols **(Right)** for Experiment 3. Congruent and incongruent trials are indicated on the *x*-axis. The separate lines illustrate conscious (solid lines) and non-conscious (dashed lines) trials. Error bars represent SEM.

All errors (8.59%) were excluded and analyzed separately (see below). Mean correct go-trial RTs were subjected to a mixed-model ANOVA, with the within-participant variable congruence (congruent, incongruent) and the between-participants variable consciousness (conscious, non-conscious). A significant main effect of congruence, *F*(1,34) = 82.71, *p* < 0.001, $\eta_{p}^{2}$ = 0.71, interacted with consciousness, *F*(1,34) = 6.21, *p* = 0.018, $\eta_{p}^{2}$ = 0.15. No main effect of consciousness was found, *F*(1,34) = 0.12. As in Experiments 1 and 2, RTs were shorter in congruent than incongruent trials for both the non-conscious (congruent: 558 ms; incongruent: 581 ms), *t*(17) = 5.43, *p* < 0.001, *d* = 1.81, and the conscious (congruent: 542 ms; incongruent: 582 ms) conditions, *t*(25) = 7.29, *p* < 0.001, *d* = 2.43. Yet, the RT congruence effect (incongruent RT minus congruent RT) was significantly larger in the conscious (40 ms) than in the non-conscious (23 ms) condition, *t*(34) = 2.49, *p* = 0.018, *d* = 0.83. As in Experiments 1 and 2, an analysis restricted to only the rating trials yielded essentially the same results. **Figure 4** (left panel) illustrates the results.

#### Error Rates (ERs)

The analysis was the same as for the RTs, with the additional within-participant variable task (go, no-go). The main effects of task, *F*(1,34) = 40.25, *p* < 0.001, $\eta_{p}^{2}$ = 0.54, and congruence, *F*(1,34) = 17.17, *p* < 0.001, $\eta_{p}^{2}$ = 0.34, also interacted with each other, *F*(1,34) = 5.12, *p* = 0.030, $\eta_{p}^{2}$ = 0.13. Only in the go trials was the ER higher in incongruent (10.62%) compared to congruent trials (5.09%), *t*(35) = 4.56, *p* < 0.001, *d* = 1.07. No such difference was found in the no-go trials, *t*(35) = 1.73, *p* = 0.093, *d* = 0.41. No other effects were found, all non-significant *F* < 2.88, all *p* > 0.099.

#### Ratings of the Chinese Symbols

The analysis was based on correct flanker-task trials. No overall bias toward negative (or positive) evaluations was found, neither in the non-conscious, *t*-test against 50%: *t*(17) = 0.90, *p* > 0.249, *d* = 0.30, *BF* = 3.81 in favor of the null hypothesis, nor in the conscious condition, *t*(17) = -0.69, *p* > 0.249, *d* = 0.23, *BF* = 4.45 in favor of the null hypothesis. A mixed-model ANOVA, with the same variables as in the ERs, revealed a main effect of congruence, *F*(1,34) = 10.95, *p* = 0.002, $\eta_{p}^{2}$ = 0.24, and an interaction between congruence and consciousness, *F*(1,34) = 10.81, *p* = 0.002, n_p_^2^= 0.24. In the non-conscious block, participants rated the symbols less often as negative in congruent (47.75%) than in incongruent (55.63%) trials, *t*(17) = 4.17, *p* = 0.001, *d* = 1.39, *BF* = 55.39 in favor of the alternative hypothesis. There was no such difference in the conscious block (congruent: 48.63%; incongruent: 48.61%), *t*(17) = -0.02, *p* > 0.249, *d* = 0.01, *BF* = 5.59 in favor of the null hypothesis. No other effects were found, all non-significant *F* < 2.41, all *p* > 0.130. The results are illustrated in **Figure 4** (right panel).

#### Distractor Awareness

Participants in the conscious condition could discriminate the distractor with above chance-level accuracy (*d’* = 1.36), *t*-test against zero: *t*(17) = 6.13, *p* < 0.001, *d* = 2.04, *BF* = 2299.58 in favor of the alternative hypothesis. Discrimination performance for participants in the non-conscious condition was not different from chance (*d’* = -0.14), *t*(17) = 1.36, *p =* 0.191, *d* = 0.45, *BF* = 2.40 in favor of the null hypothesis (corresponding to no substantial evidence). No difference in the response bias was found between the conscious condition (β = 1.22) and non-conscious condition (β = 0.99), *t*(34) = 1.40, *p =* 0.172, *d* = 0.47, *BF* = 3.04 in favor of the null hypothesis.

### Discussion

Experiment 3 replicated Experiment 2 and confirmed that the block order was not responsible for the differences between conscious and non-conscious blocks. Again, we found a larger proportion of negative ratings after incongruent than congruent target-distractor pairs. However, this result was only apparent in the non-conscious block, not in the conscious block. Furthermore, we found slower RTs in incongruent than congruent trials, and this congruence effect was larger in the conscious than in the non-conscious condition.

One notable difference to Experiment 2 was the absence of an influence of task (go versus no-go trials) on ratings. This could be explained by the smaller statistical power of the mixed-model design compared with the within-participant design of Experiment 2. Furthermore, the Bayesian *t*-test of this effect in Experiment 2 already indicated that evidence of this effect was not entirely conclusive, so that our failure to replicate this effect might reflect data insensitivity.

## General Discussion

We found more affect misattributions following non-conscious distractors than conscious distractors. Only following non-conscious incongruent distractors but not after conscious incongruent distractors, the proportion of negative ratings of an otherwise neutral symbol increased. This affective priming effect was likely due to the participants’ lacking awareness of the non-conscious distractors as the true affect-origins, as indicated by the participants’ unawareness of the non-conscious distractors. The unawareness of the true affect-origins probably facilitated a misattribution of the conflict-elicited affective responses to the neutral symbols. As such, the results support a misattribution explanation of the affective priming effect on neutral symbols (Payne et al., 2010). In fact, because we found more response priming with the conscious distractors than with the non-conscious distractors, the full data pattern amounted to a double dissociation between response priming (stronger by conscious distractors) and affective priming (stronger by non-conscious distractors). This means that simply more response priming by the non-conscious distractors was not responsible for their stronger affective priming effect. Instead, the unawareness of the non-conscious distractors somehow facilitated the affective priming effect and the awareness of the distractors in the conscious conditions somehow undermined the affective priming effect. For example, participants’ awareness of the distractor-target conflict might have allowed them to correctly attribute their feelings to this conflict. This in turn could have undermined any misattributions of the same feelings to the Chinese symbols. Alternatively, awareness of the distractor-target conflict could have also allowed the participants to actively suppress the spread of their feelings to the evaluation of the Chinese symbols because the Chinese symbols were now clearly recognized as not being responsible for the currently pertaining feeling.

To our surprise, however, the results in the conscious condition were relatively extreme: We found no evidence for affective priming of the symbol ratings following conscious distractors whatsoever. This is surprising because a prior Stroop study found affective priming with conscious Stroop stimuli (e.g., Fritz and Dreisbach, 2013). Several reasons for this difference are conceivable. First of all, conflict in Stroop tasks is not the same as in flanker tasks. Some sources of conflict in the Stroop task are not effective in the flanker task. For example, only in the Stroop but not in the flanker task, incongruent trials created higher attentional demands during long-term memory retrieval (Keele, 1972; MacLeod, 1991). The reason is that only in the Stroop task, colors (as targets) and color names (as distractors) were used, so that long-term memory associations between irrelevant color names and responses could have contributed to interference in incongruent conditions. No such long-term memory associations would have been at work in the incongruent flanker conditions of the present study. Another difference between Stroop task and our flanker task concerns the degrees of task conflict in incongruent conditions. In an incongruent Stroop trial, the distractors had the potential to elicit task conflict between the distractor words that tend to elicit their (not instructed) reading and the target colors that, per instruction, had to be named (see MacLeod and MacDonald, 2000; Goldfarb and Henik, 2007). This kind of task interference was absent in the incongruent conditions of the flanker task that we used here. If either of the two aforementioned Stroop-specific conflict sources depends on an awareness of the distractors, the presence of long-term memory based conflict or of the presence of task conflict in the incongruent Stroop trials of Fritz and Dreisbach (2013) could have fostered an affective priming effect of the conscious distractors on symbol ratings in that study. By the same token, the absence of the same conflict sources in the flanker task could have prevented some conflict-dependent affect misattributions based on the present study’s conscious distractors.

Moreover, in the present experiments, conflict was elicited by a target-distractor relation that could follow a different time course than the Stroop interference. In the present study, we used distractor-target sequences that were presented 1 s before and, thus, well ahead of the neutral symbols. In contrast, the interval between Stroop stimuli and neutral symbols in the study of Fritz and Dreisbach (2013) was 400 ms which is, relatively short. Moreover, Fritz and Dreisbach (2015) showed that with increasing interval between conflicting Stroop stimulus and to-be-rated neutral stimulus, the effect of incongruent Stroop stimuli on the ratings of neutral stimuli decreased. Since our procedure used an interval between the target and the neutral symbol of 1 s, we might have missed out on time-dependent and more fleeting affective priming effects in the conscious condition. However, unless one assumes that the affective priming effect is more robust and less fleeting in the non-conscious condition, it is not clear why the time interval would have selectively influenced the affective priming effect in the conscious and not in the non-conscious condition. In other words, our interpretation of a lack of awareness of the true affect-origin as a supportive factor for affect misattributions in non-conscious conditions would still be plausible and not be challenged by this possibility.

## Conclusion

For many applications, such as for rational decisions based on evaluations and for attitudes toward objects and persons, it is important to understand the favorable side conditions of correct and incorrect affect attributions. This is so important because the affective responses toward objects and persons shape and trigger human attitudes toward these objects and persons, and ultimately determine actions, such as how much another person is pitied and helped. Here, we have identified non-conscious processing of affect-origins as a favorable side condition for affect misattributions away from their true affect-origins and toward other objects. This finding implies that sufficient awareness of the true affect-origins is helpful for correct affect attributions and, thus, could be a precondition for rational human action (see also Topolinski and Strack, 2009).

## Author Contributions

FG and UA developed the study concept and the study design. FG performed the data collection, data analysis and interpretation under the supervision UA. FG drafted the manuscript, both SK and UA substantially contributed to the interpretation of the data, and provided many important critical revisions. All authors approved the final version of the manuscript for submission. All authors agree to be accountable for all aspects of the work in ensuring that questions related to the accuracy or integrity of any part of the work are appropriately investigated and resolved.

## Conflict of Interest Statement

The authors declare that the research was conducted in the absence of any commercial or financial relationships that could be construed as a potential conflict of interest.

## References

[B1] Ansorge U. (2004). Top-down contingencies of nonconscious priming revealed by dual-task interference. *Q. J. Exp. Psychol. A* 57 1123–1148. 10.1080/02724980343000792 15370519

[B2] Arnold M. B. (1960). *Emotion and Personality.* New York, NY: Columbia University Press.

[B3] Brainard D. H. (1997). The psychophysics toolbox. *Spat. Vis.* 10 433–436. 10.1163/156856897X00357 9176952

[B4] Casasanto D. (2009). Embodiment of abstract concepts: good and bad in right-and left-handers. *J. Exp. Psychol.* 138 351–367. 10.1037/a0015854 19653795

[B5] Dienes Z. (2011). Bayesian versus orthodox statistics: which side are you on? *Perspect. Psychol. Sci.* 6 274–290. 10.1177/1745691611406920 26168518

[B6] Dienes Z. (2014). Using Bayes to get the most out of non-significant results. *Front. Psychol.* 5:781 10.3389/fpsyg.2014.00781 PMC4114196 25120503

[B7] Eriksen B. A. Eriksen C. W. (1974). Effects of noise letters upon the identification of a target letter in a nonsearch task. *Percept. Psychophys.* 16 143–149. 10.3758/BF03203267

[B8] Faul F. Erdfelder E. Buchner A. Lang A.-G. (2009). Statistical power analyses using G^∗^Power 3.1: tests for correlation and regression analyses. *Behav. Res. Methods* 41 1149–1160. 10.3758/BRM.41.4.1149 19897823

[B9] Fenske M. J. Raymond J. E. Kessler K. Westoby N. Tipper S. P. (2005). Attentional inhibition has social-emotional consequences for unfamiliar faces. *Psychol. Sci.* 16 753–758. 10.1111/j.1467-9280.2005.01609.x 16181435

[B10] Fritz J. Dreisbach G. (2013). Conflicts as aversive signals: conflict priming increases negative judgments for neutral stimuli. *Cogn. Affect. Behav. Neurosci.* 13 311–317. 10.3758/s13415-012-0147-1 23307475

[B11] Fritz J. Dreisbach G. (2015). The time course of the aversive conflict signal. *Exp. Psychol.* 62 30–39. 10.1027/1618-3169/a000271 25270558

[B12] Gawronski B. Ye Y. (2014). What drives priming effects in the affect misattribution procedure? *Pers. Soc. Psychol. Bull.* 40 3–15. 10.1177/0146167213502548 23982152

[B13] Goldfarb L. Henik A. (2007). Evidence for task conflict in the Stroop effect. *J. Exp. Psychol.* 33 1170–1176. 10.1037/0096-1523.33.5.1170 17924815

[B14] Gratton G. Coles M. G. Sirevaag E. J. Eriksen C. W. Donchin E. (1988). Pre-and poststimulus activation of response channels: a psychophysiological analysis. *J. Exp. Psychol.* 14 331–344. 10.1037/0096-1523.14.3.331 2971764

[B15] Green D. M. Swets J. A. (1966/1974). *Signal Detection Theory and Psychophysics (A Reprint, with Corrections of the Original 1966 Ed.).* Huntington, NY: Robert E. Krieger Publishing Co.

[B16] Jeffreys H. (1961). *The Theory of Probability* 3rd Edn Oxford: Oxford University Press.

[B17] Keele S. W. (1972). Attention demands of memory retrieval. *J. Exp. Psychol.* 93 245–248. 10.1037/h0032460 5025731

[B18] Klotz W. Neumann O. (1999). Motor activation without conscious discrimination in metacontrast masking. *J. Exp. Psychol.* 25 976–992. 10.1037/0096-1523.25.4.976

[B19] Kouider S. Dupoux E. (2004). Partial awareness creates the “illusion” of subliminal semantic priming. *Psychol. Sci.* 15 75–81. 10.1111/j.0963-7214.2004.01502001.x 14738512

[B20] MacLeod C. M. (1991). Half a century of research on the Stroop effect: an integrative review. *Psychol. Bull.* 109 163–203. 10.1037/0033-2909.109.2.163 2034749

[B21] MacLeod C. M. MacDonald P. A. (2000). Interdimensional interference in the Stroop effect: uncovering the cognitive and neural anatomy of attention. *Trends Cogn. Sci.* 4 383–391. 10.1016/S1364-6613(00)01530-8 11025281

[B22] Murphy S. T. Zajonc R. B. (1993). Affect, cognition, and awareness: affective priming with optimal and suboptimal stimulus exposures. *J. Pers. Soc. Psychol.* 64 723–739. 10.1037/0022-3514.64.5.723 8505704

[B23] Neumann O. Klotz W. (1994). “Motor responses to nonreportable, masked stimuli: where is the limit of direct parameter specification,” in *Attention and Performance XV: Conscious and Nonconscious Information Processing* eds Umiltà C. Moscovitch M. (Cambridge, MA: MIT Press) 123–150.

[B24] Oikawa M. Aarts H. Oikawa H. (2011). There is a fire burning in my heart: the role of causal attribution in affect transfer. *Cogn. Emot.* 25 156–163. 10.1080/02699931003680061 21432663

[B25] Ortony A. Clore G. L. Collins A. (1990). *The Cognitive Structure of Emotions.* Cambridge, MA: Cambridge University Press.

[B26] Payne B. Hall D. Cameron C. Bishara A. (2010). A process model of affect misattribution. *Pers. Soc. Psychol. Bull.* 36 1397–1408. 10.1177/0146167210383440 20837777

[B27] Payne B. K. Cheng C. M. Govorun O. Stewart B. D. (2005). An inkblot for attitudes: affect misattribution as implicit measurement. *J. Pers. Soc. Psychol.* 89 277–293. 10.1037/0022-3514.89.3.277 16248714

[B28] Pelli D. G. (1997). The VideoToolbox software for visual psychophysics: transforming numbers into movies. *Spat. Vis.* 10 437–442. 10.1163/156856897X00366 9176953

[B29] Raymond J. E. Fenske M. J. Tavassoli N. T. (2003). Selective attention determines emotional responses to novel visual stimuli. *Psychol. Sci.* 14 537–542. 10.1046/j.0956-7976.2003.psci_1462.x 14629683

[B30] Reingold E. M. Merikle P. M. (1988). Using direct and indirect measures to study perception without awareness. *Percept. Psychophys.* 44 563–575. 10.3758/BF03207490 3200674

[B31] Rouder J. N. Speckman P. L. Sun D. Morey R. D. Iverson G. (2009). Bayesian t-tests for accepting and rejecting the null hypothesis. *Psychon. Bull. Rev.* 16 225–237. 10.3758/PBR.16.2.225 19293088

[B32] Schachter S. Singer J. E. (1962). Cognitive, social, and physiological determinants of emotional state. *Psychol. Rev.* 69 379–399. 10.3758/BF03193692 14497895

[B33] Schmidt T. Vorberg D. (2006). Criteria for unconscious cognition: three types of dissociation. *Percept. Psychophys.* 68 489–504. 10.3758/BF03193692 16900839

[B34] Schouppe N. Braem S. De Houwer J. Silvetti M. Verguts T. Ridderinkhof K. R. (2015). No pain, no gain: the affective valence of congruency conditions changes following a successful response. *Cogn. Affect. Behav. Neurosci.* 15 251–261. 10.3758/s13415-014-0318-3 25183556

[B35] Schwarz N. (1990). “Feelings as information: informational and motivational functions of affective states,” in *Handbook of Motivation and Cognition* Vol. 2 eds Higgins E. T. Sorrentino R. M. (New York, NY: Guilford Press) 527–561.

[B36] Schwarz W. Mecklinger A. (1995). Relationship between flanker identifiability and compatibility effect. *Percept. Psychophys.* 57 1045–1052. 10.3758/BF03205463 8532494

[B37] Stanislaw H. Todorov N. (1999). Calculation of signal detection theory measures. *Behav. Res. Methods Instrum. Comput.* 31 137–149. 10.3758/BF03207704 10495845

[B38] Tapia E. Breitmeyer B. G. Shooner C. R. (2010). Role of task-directed attention in nonconscious and conscious response priming by form and color. *J. Exp. Psychol.* 36 74–87. 10.1037/a0017166 20121296

[B39] Topolinski S. Strack F. (2009). The architecture of intuition: fluency and affect determine intuitive judgments of semantic and visual coherence and judgments of grammaticality in artificial grammar learning. *J. Exp. Psychol.* 138 39–63. 10.1037/a0014678 19203169

[B40] van Steenbergen H. Band G. P. Hommel B. (2010). In the mood for adaptation how affect regulates conflict-driven control. *Psychol. Sci.* 21 1629–1634. 10.1177/0956797610385951 20943936

[B41] Weiner B. (1986). *An Attributional Theory of Motivation and Emotion.* New York, NY: Springer-Verlag.

[B42] Zajonc R. B. (1968). Attitudinal effects of mere exposure. *J. Pers. Soc. Psychol.* 9 1–27. 10.1037/h00258485667435

